# Bowel wall thickness measured by MRI is useful for early diagnosis of bowel endometriosis

**DOI:** 10.1007/s00330-023-09795-7

**Published:** 2023-07-27

**Authors:** Yunxi Zheng, Shouxin Gu, Jingyao Ruan, Xiaofang Yi, Congjian Xu

**Affiliations:** 1 Department of Gynecology, Obstetrics and Gynecology Hospital, Fudan University, Shanghai, 200011 People’s Republic of China; 2 Department of Obstetrics and Gynecology of Shanghai Medical School, Fudan University, Shanghai, 200011 People’s Republic of China; 3 Shanghai Key Laboratory of Female Reproductive Endocrine Related Diseases, Shanghai, 200011 People’s Republic of China; 4 Department of Radiology, Obstetrics and Gynecology Hospital, Fudan University, Shanghai, 200011 People’s Republic of China

**Keywords:** Endometriosis, Magnetic resonance imaging, Diagnosis, Predictive value, ROC Curve

## Abstract

**Objective:**

To evaluate MRI features of bowel endometriosis (BE) and verify its clinical significance compared with pathological diagnosis.

**Materials and methods:**

Since 2018, patients clinically diagnosed with deep endometriosis (DE) and planned to undergo surgery were enrolled prospectively. MRI parameters including traction, thickening sign of the rectum, obliteration of the Douglas Pouch, sign of adenomyosis, and pelvic adhesion were extracted. Uni- and multi-variate analyses were performed to explore their association with pathological diagnosis of BE. ROC curve was utilized to ascertain the appropriate cutoff value for predicting the presence and assessing the severity of BE.

**Results:**

A total of 226 patients with DE were recruited, and 154 BE cases were pathologically confirmed. Logistic regression analysis revealed that thickness of the rectal wall, traction sign of the rectum, and obliteration of the Douglas Pouch were independent factors to predict the presence of BE with the OR 1.59 (95% CI: 1.29–1.96), 0.24 (95% CI: 0.09–0.67), and 0.17 (95% CI: 0.07–0.40), respectively (*p* all < 0.01). A cutoff value of 6.0 mm for the thickness of rectal wall resulted in the highest predictive value of BE (specificity: 90.3%; sensitivity: 78.6%). For patients with measured thickness of the rectal wall over 6.0 mm, 72.1% (93/129) was confirmed BE with lesions infiltrated more than muscular layer.

**Conclusion:**

This prospective study indicates that based on precise definition of visualized features on MRI images, BE could be recognized pre-operatively. DE patients with thickness of rectal wall exceeding 6.0 mm have a greater probability of BE.

**Clinical relevance statement:**

Based on precise definition of visualized features and accurate measurement on MRI images, bowel infiltrating among deep endometriosis patients could be recognized pre-operatively.

**Key Points:**

• *Precise definition of measurable MRI parameters made it possible for early detection of bowel endometriosis.*

• *Thickening sign, traction sign of the rectum, and obliteration of the Douglas Pouch were typical radiological indicators for bowel endometriosis.*

• *Bowel involvement is more sensitive to be detected among pelvic deep endometriosis patients with the thickness of the rectal wall over 6.0 mm.*

**Supplementary information:**

The online version contains supplementary material available at 10.1007/s00330-023-09795-7.

## Introduction

Deep endometriosis (DE) is considered the most severe type of endometriosis, which significantly affects women’s reproductive health. Bowel endometriosis (BE), accounting for about 5–12% of DE[1], is defined as deep endometriotic lesions infiltrated deeper than serosal layer of the rectum [2]. Patients may experience severe dysmenorrhea, dyschezia, and dyspareunia, which impacts their quality of life significantly and brings high social costs [3, 4].

Delay of diagnosis is a common situation for BE, due to insufficient awareness of symptoms and lacked experience among junior doctors [5–7], which subsequently leads to delayed practice of effective interventions and substantial medical burdens [4]. ESHRE/ESGE recommends the management of complicated deep endometriosis cases by multidisciplinary team (MDT) collaboration that includes colorectal surgeons [8]. However, even when working alongside colorectal surgeons, gynecologists could not make the correct decision regarding conservative or radical resection of bowel endometriosis if the extent of endometriosis was not predicted preoperatively. Studies indicate a potential 10% occurrence of overall postoperative complications for BE cases, even in specialized endometriosis centers [9], and inadequate elimination of endometriotic lesions leads to a higher recurrence rate [10]. Nevertheless, the overestimation of BE based on immature radiological impressions may lead to unnecessary segmental resection of the bowel. Therefore, early diagnosis and accurate judgment of BE should be a top priority and the “first” step in long-term management of BE.

Pelvic magnetic resonance imaging (MRI) serves as a non-invasive technique and has been widely used to evaluate pelvic mass. Previous studies revealed the optimal efficacy of MRI in tumor staging for rectal cancers due to the accurate measurement of the infiltrating depth to the rectal wall [11, 12]. Although MRI has been recommended by the European Society of Urogenital Radiology (ESUR) as the first-line examination for endometriosis [13], it is still unclear whether MRI really “visualize” the infiltrating depth of pelvic DE lesion. Currently, most of the radiological criteria for diagnosis of DE are subjective, with no quantitative parameters defining DE-related MRI features. Further, BE is manifested as severe adhesion, infiltration, or fibrosis of bowel segments, and presents more difficulty in diagnosis and differentiation. Therefore, early detection of BE is a great challenge for gynecologists and radiologists.

Even with MDT, either underestimation or overestimation of BE may lead to inadequate decision-making during the surgery. Therefore, to precisely judge BE among women with suspected pelvic DE, we carried out the current study. We aimed to screen and quantify characteristic MRI features of BE and assess its clinical significance for early detection of BE.

## Material and methods

### Patients’ enrollment

**Fig. 1:**
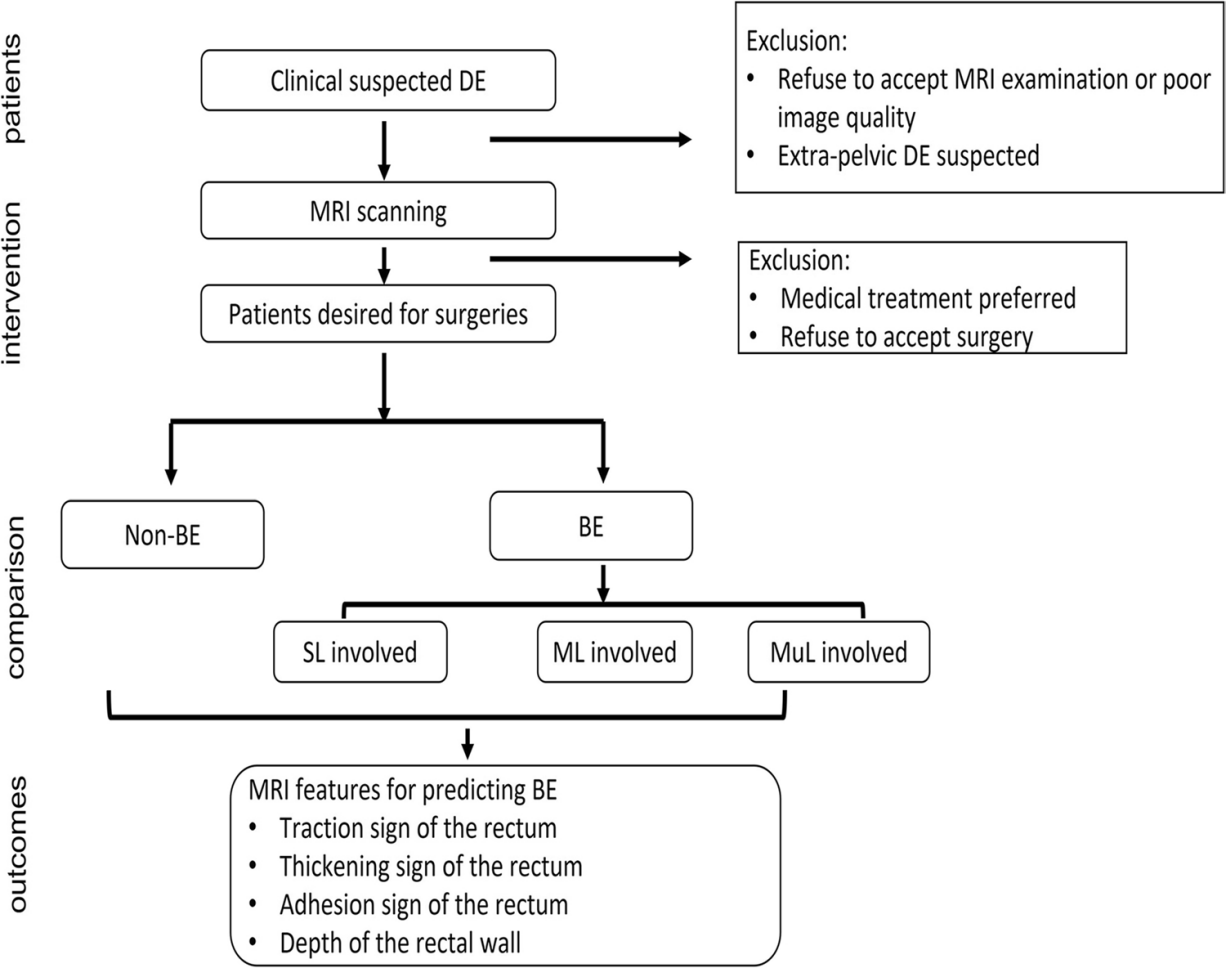
Flowchart of recruitment for participants. DE, deep endometriosis; BE, bowel endometriosis; non-BE, deep endometriosis without bowel involvement; SL, sub-serosal layer of rectum wall; ML, muscular layer of rectum wall; MuL, mucous layer of rectum wall

This prospective study was carried out under the approval of the Ethics Committee of the Obstetrics and Gynecology Hospital of Fudan University (Ethics Number: 2016–06) in accordance of the Declaration of Helsinki. Consecutive patients clinically diagnosed with DE and decided to undergo laparoscopic surgery were enrolled since August 1, 2018 (Fig. 1).

### Inclusion criteria

1. DE was clinically suspected based on clinical history, including progressively severe dysmenorrheal, dyschezia, or dyspareunia, with visual analog scale score more than 5 points [3].
2. Physical examination carried out by the same gynecological medical team, with the sense of frozen pelvis and/or tender nodule detected at the Douglas Pouch.
3. Preoperative MRI examination was performed within 1 month before the surgery, and high-quality MRI images were available.
4. Complete removal of the endometriotic lesions would be carried out by laparoscopic surgery.

### Exclusion criteria

1. No MRI examination record within 1 month pre-operatively, or only poor-quality MRI images were available.
2. Conservative treatment was preferred for the lesions.
3. Reluctant to accept relatively radical surgery to remove lesions.

### MR image acquisition and processing

The patient was positioned supine without bowel preparation prior to the MRI examination. Neither buscopan/glucagon were given, nor rectal/vaginal gel was administered. Pelvic MR images were acquired on a 1.5-T MRI scanner (Siemens Medical), and scanning parameters are shown in Supplementary Table 1.

Axial and sagittal dynamic contrast-enhanced sequences were acquired after intravenous injection of Gd-DTPA at a dose of 0.2 mL/kg of body weight (Omniscan, GE Healthcare) and at the rate of 2 mL/s. All images were processed via picture archiving and communication system.

### Precise definition for MRI indicators

**Fig. 2:**
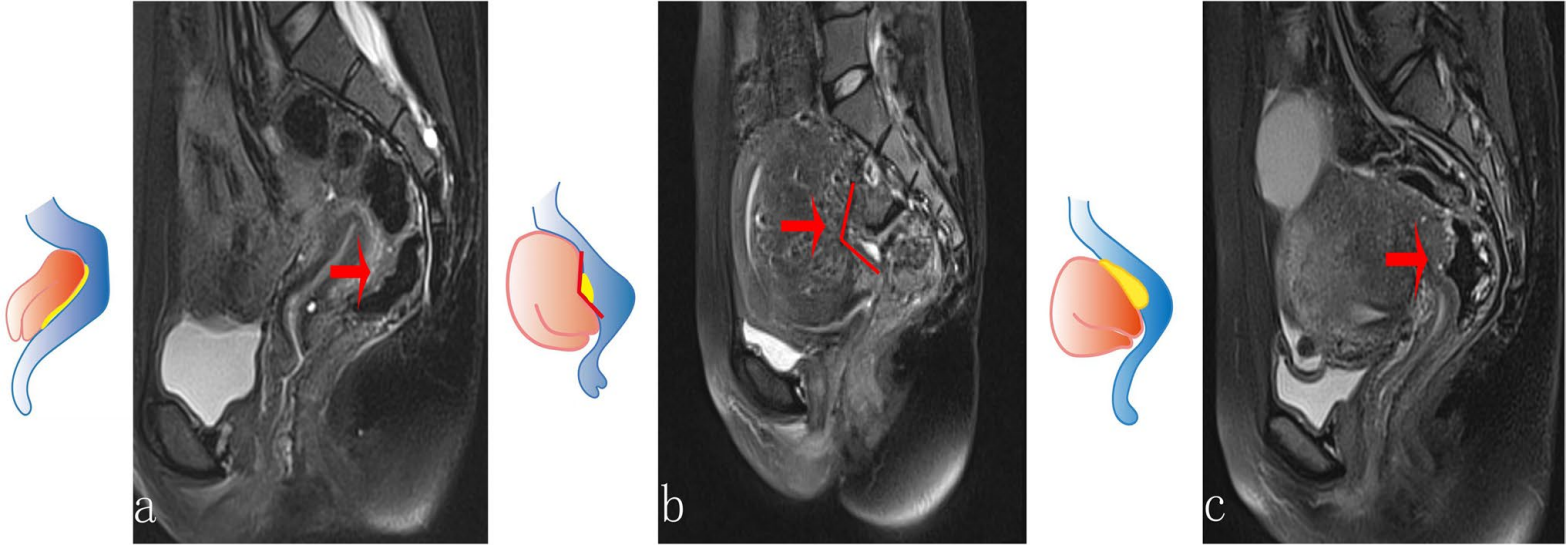
Major features in pelvic MRI examination and the corresponding schematic drawings. **a** Obliteration of the Douglas Pouch (red arrow), anatomic structure of posterior cul-de-sac could not be detected on MRI images, with extremely adhesion sign between the anterior rectum and the posterior uterus, or ovary; **b** traction sign of the rectum (red arrow), natural morphology of the rectum was instead by stiff appearance, which lead to the angulated changes of selected segmental rectum; **c** thickening sign of the rectal wall: the increasing trend of the rectal wall thickness

**Fig. 3:**
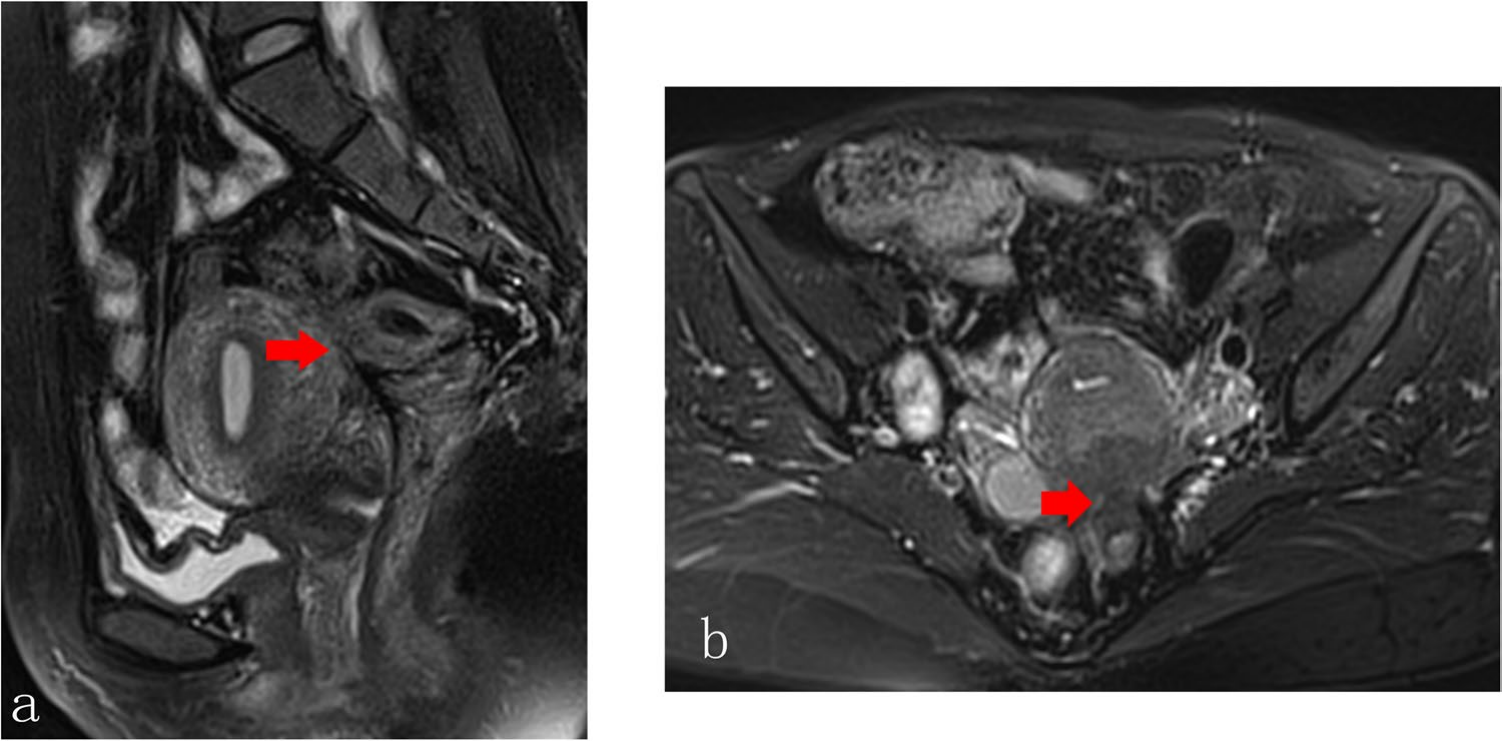
Pelvic adhesion sign. **a** The Douglas Pouch was clearly visible, while severe adhesion was detected in the pelvis (red arrow); **b** the posterior uterus and the anterior upper part of the rectum was fused(red arrow)

**Fig. 4:**
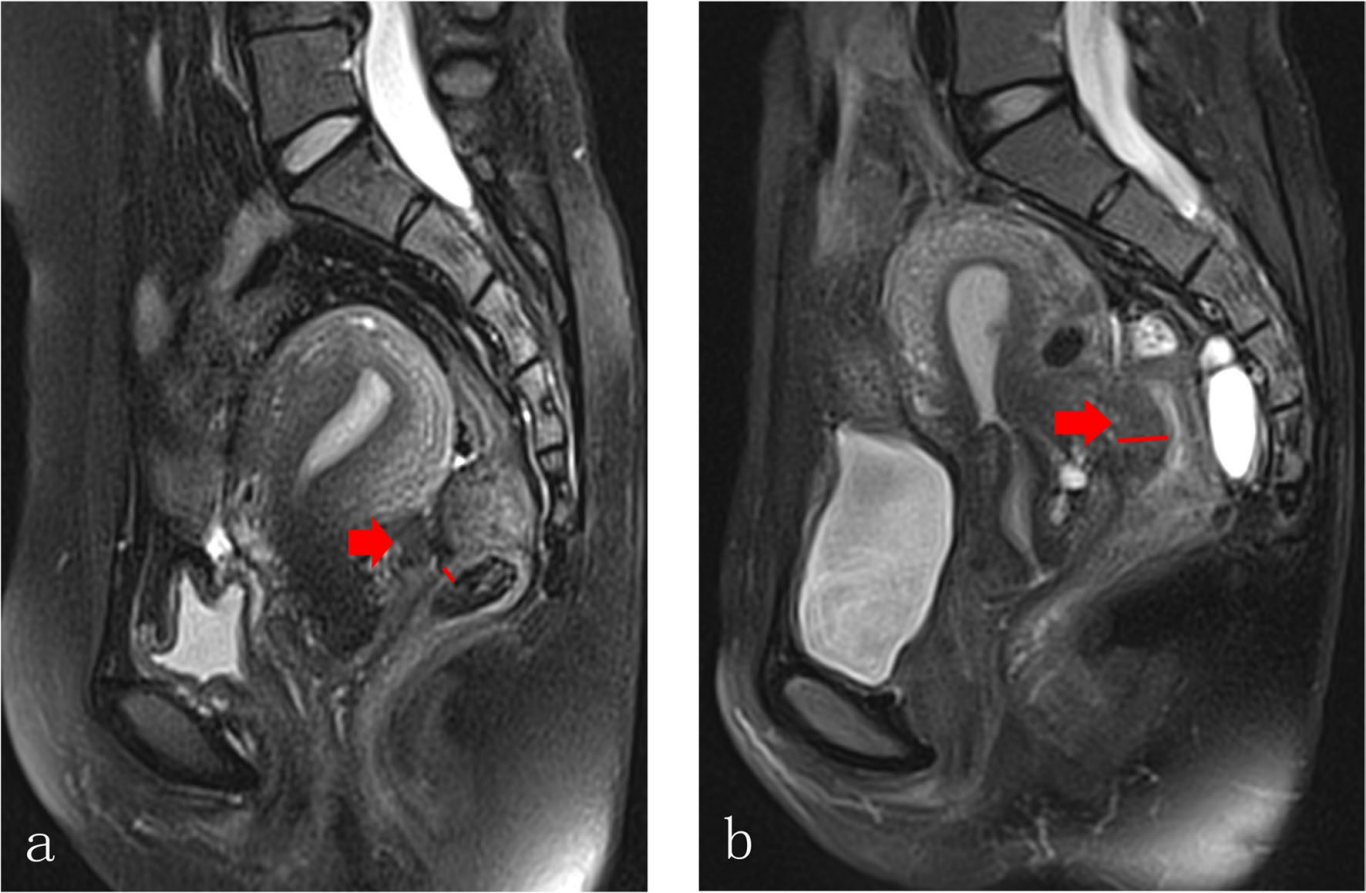
Measurement for the thickness of the rectal wall. Based on the sagittal plane of T2-FS images, the maximum part of the involved rectum was measured from serosal layer to the mucosal layer of the rectal wall (from outer to inner layer of the rectal wall) by caliper. **a** Deep endometriosis lesion (red arrow) was located in the Douglas Pouch without infiltrating to the rectal wall, the thickness of the rectal wall was 3.1 mm (red short line); **b** bowel endometriosis (red arrow) was presented with the maximum thickness of the rectal wall was 16.2 mm (redline)

A novel protocol of visualization method for delineating the pelvic anatomic landmarks was developed. Boundaries for the pelvic regions were described as Douglas Pouch, rectum, and anal verge. Graphic features of MRI were mainly extracted on the standard sagittal section of T2-fat suppression sequence (T2-FS), supplemented with other scanning signs, including thickening, traction sign of the rectum, obliteration of the Douglas Pouch, and pelvic adhesion (Figs. 2 and 3). The location of the rectum was defined in accordance of diagnosis and radiological examination guidelines for rectal cancers [14, 15]. Length distance from the anal verge to the inferior margin lowest part of the endometriotic lesion (mm) was measured using the sum of multiple straight lines on sagittal plane. The rectum was divided into three segments according to the distance between the lowest part of the lesion to the anus: upper (> 10 cm), middle (5–10 cm), and lower (< 5 cm). Traction sign was founded when natural morphology of the rectum was replaced by stiff lesion, leading to the angulated changes of involved segments [16]. Given that the rectum is the most common place for bowel endometriosis; therefore, the maximum thickness of the rectal wall was measured. We defined the thickness of the rectal wall as the maximum measured thickness from serosal layer to the mucosal layer of the rectal wall (from outer to inner layer), with measurements carried out by caliper on the sagittal plane of T2-FS images (Fig. 4). For patients with multiple endometriotic lesions, the largest lesion was selected for analysis.

### Radiological diagnostic criteria for DE

**Fig. 5:**
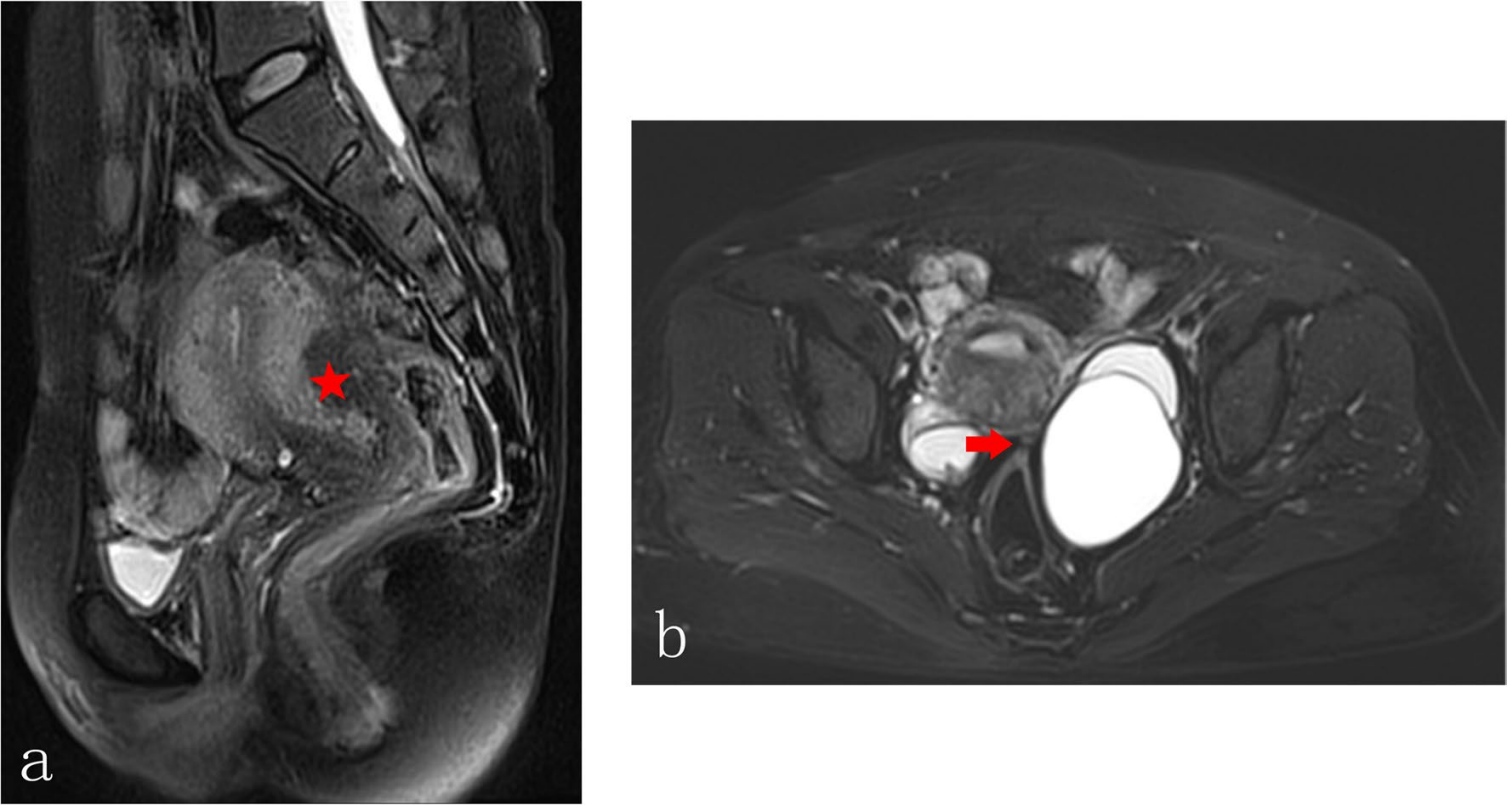
Radiological diagnosis for deep endometriosis. **a** The sagittal plane showed the irregular solid mass (red star) located in the posterior pelvis, with extrinsic adenomyosis. The rectal cavity was compressed by the solid mass. **b** Patient suffered bilateral endometriomas and deep endometriosis in the left uterosacral ligament (red arrow), the transverse plane showed retroversion fixation of uterus

Radiological diagnostic criteria for DE are as follows: (1) irregular solid mass located in the posterior pelvis, and presented as hyper-intensity on T2WI images and iso-intensity on T1WI images; (2) disappearance of fatty space between posterior uterus and anterior rectal wall; (3) other indirect signs or accompanying signs including compression of rectal cavity, uterine serosal plaque with low signal intensity similar to fibrous tissue, retroversion fixation of uterus, bilateral asymmetrical thickening of uterosacral ligaments, and elevated vaginal fornix [17, 18]. One or more radiological sign was presented, and DE was radiologically concerned (Fig. 5).

Two senior radiologists (Gu S and Ma F) reviewed the MRI images independently. For inter-observer discrepancies in the evaluation of data extraction, the consensus was achieved by inviting a third senior radiologist (Zhang G).

### Intervention

All patients underwent laparoscopic surgery and diagnosis of DE was confirmed by pathological examination. The confirmation of BE requires infiltrating of endometriotic tissue (endometrial gland and stroma cells) beyond the serosal layer of the rectal wall [2]. All patients were assigned into two groups according to their post-operative pathological diagnosis: BE and non-BE. Case-by-case analysis was carried out to compare MRI images and pathological findings (Supplementary Fig. 1).

### Statistical analysis

Quantitative data were presented as mean ± standard deviation, and qualitative data were expressed as the number of cases and percentage. Univariate analysis was performed by SPSS (version 21.0; IBM Inc.). For continuous variables, student *T*-test was carried out for normal distribution data. Pearson’s chi-square test (or Fisher’s exact test) was used for the categorical variables between two groups. Logistic regression model was used for multivariate analysis, to analyze the independent factors for the prediction of bowel endometriosis. *p* < 0.05 was regarded as statistically significant. The cutoff values for bowel endometriosis were calculated based on the maximum Youden index (YI) of the ROC curve.

## Results

### Patients’ characteristics

A total of 366 suspected DE patients who decided to receive surgical treatment were recruited. Referring to the inclusive and exclusive criteria, 226 patients were enrolled in the current study. The lesion was precisely delineated by MRI, among 186 patients who were diagnosed with BE by MRI, 154 of them were confirmed by pathological examination. Dysmenorrhea, dyschezia, and dyspareunia were the most common complaints. Out of the 226 patients, 25.7% (58/226) were recurrent cases, with 34.5% (20/58) experienced more than two endometriosis-related surgeries. Prior to the current surgery, 22.1% (50/226) of patients had tried and failed conservative medical treatment, including GnRH-a injection and/or oral contraceptives (Table 1).

**Table 1:** Clinical features of the study population

Variables	BE (*N* = 154)	Non-BE (*N* = 72)	*p* value
Age (year)	36.9 ± 6.9	35.9 ± 9.5	0.36
BMI (kg/m^2^)	20.2 ± 6.3	20.2 ± 6.4	0.96
Dysmenorrhea	127 (82.5%)	55 (76.4%)	0.28
Dyspareunia	37 (24.0%)	12 (16.7%)	0.14
Dyschezia	96 (68.2%)	34 (47.2%)	0.03
Previous endometriosis-related surgery	43 (27.9%)	15 (20.8%)	0.26
Ovarian cystectomy	35	11	
Hysterectomy	2	1	
Hysterectomy + cystectomy	2	1	
Excision of endometriosis lesions	4	4	
Medical treatment	34 (22.1%)	16 (22.2%)	0.98
GnRH-a	23	13	
Oral contraceptives	5	2	
Combined conservative treatments	25	7	

*BE* bowel endometriosis; non-BE deep endometriosis without bowel involvement

### Predictive factors of bowel endometriosis based on MRI images

Traction sign of the rectum, obliteration of the Douglas Pouch, and sign of pelvic adhesion were typical morphological alterations among BE patients with the detection rate of 71.4% (110/154), 81.8% (126/154), and 97.4% (150/154), respectively, but their occurrence was notably lower in non-BE patients at 11.1% (8/72), 23.6% (17/72), and 69.4% (50/72) (*p* < 0.01, all) (Table 2). Thickness of the rectal wall was notably increased among BE patients at an average value of 8.43 ± 0.33 mm compared to non-BE counterparts with a thickness of 3.68 ± 0.24 (*p* < 0.01). More than half of the DE patients with lesions in the posterior pelvis suffered adenomyosis as well. Further, logistic regression analysis confirmed thickness of the rectal wall, traction sign of the rectal wall, and obliteration of the Douglas Pouch, as independent radiological changes associated with BE, with the OR 1.59 (95% CI: 1.29–1.96), 0.24 (95% CI: 0.09–0.67), and 0.17 (95% CI: 0.07–0.40), respectively (*p* all < 0.01) (Table 2).

**Table 2:** Univariate analysis and logistic regression analysis of MRI parameters in predicting bowel involvement

	Uni-variate analysis			Multi-variate analysis		
	BE (*N* = 154)	Non-BE (*N* = 72)	*p* value	OR	95% CI	*p* value
Thickness of the rectal wall (mm)	8.43 ± 0.33	3.68 ± 0.24	< 0.01	1.59	1.29–1.96	< 0.01
Traction sign of the rectum	110 (71.4%)	8 (11.1%)	< 0.01	0.24	0.09–0.67	< 0.01
Obliteration of the Douglas Pouch	126 (81.8%)	17 (23.6%)	< 0.01	0.17	0.07–0.40	< 0.01
Combined with adenomyosis	103 (66.9%)	40 (55.6%)	0.09	1.60	0.65–3.91	0.30
Sign of pelvic adhesion	150 (97.4%)	50 (69.4%)	< 0.01	0.33	0.08–1.41	0.14

*BE* bowel endometriosis; non-BE deep endometriosis without bowel involvement

### Sub-group analysis for infiltrating depth of BE

**Fig. 6:**
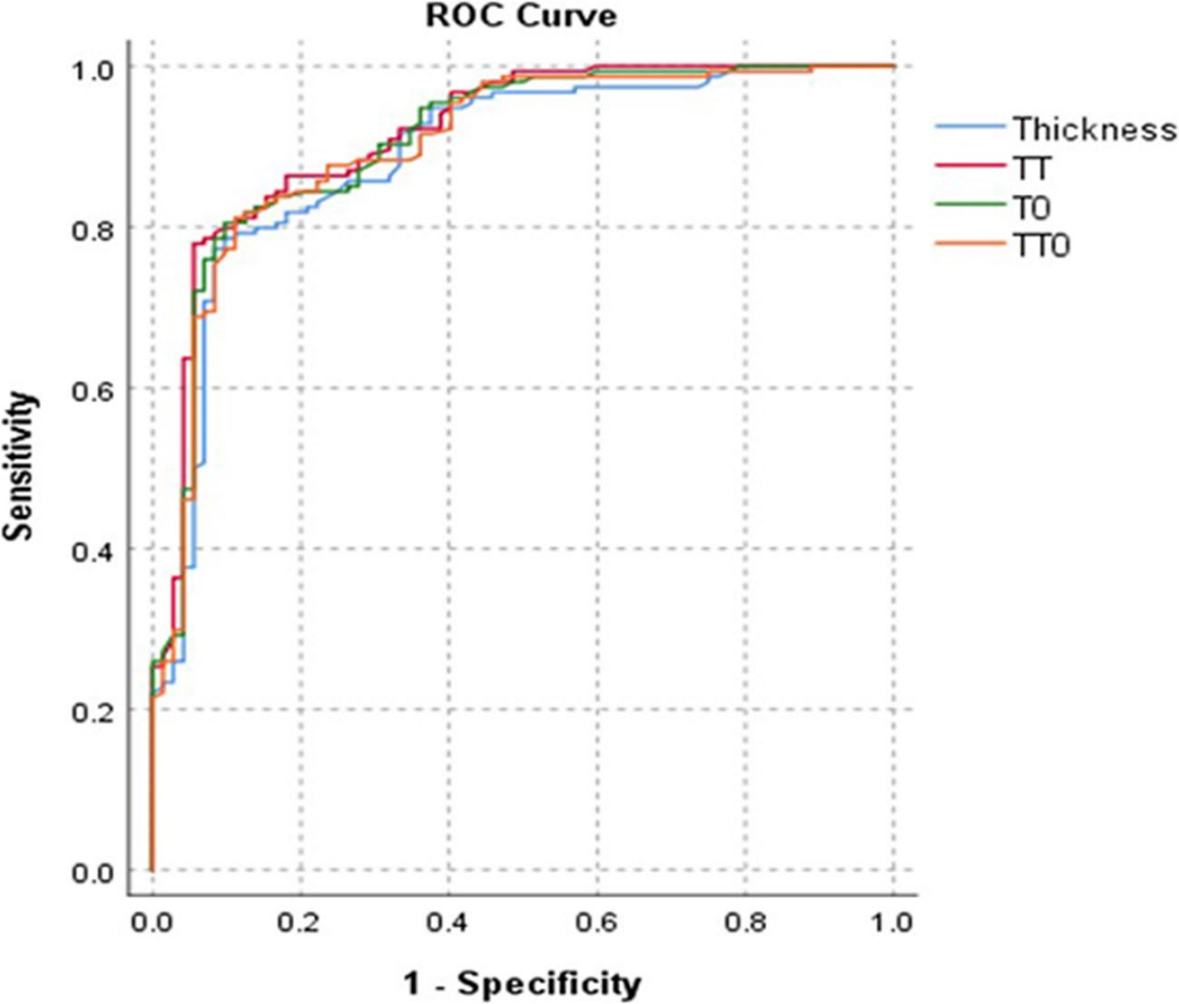
Relationship between the MRI-related parameters and the bowel involvement. ROC curve for identifying bowel endometriosis based on the thickness of the rectal wall, traction sign, and obliteration of the Douglas Pouch. AUC was 0.89 (95% CI: 0.84–0.94), 0.92 (95% CI: 0.88–0.96), 0.91 (95% CI: 0.86–0.95), and 0.90 (95% CI: 0.86–0.95), for thickness only, TT, TO, and TTO respectively. The cutoff value for thickness of rectal was 6.0 mm for predicting bowel involvement (sensitivity: 78.6%; specificity: 90.9%). TT, thickness of the rectal wall and traction sign of the rectum; TO, thickness of the rectal wall and obliteration of the Douglas Pouch; TTO, thickness of the rectal wall, traction sign of the rectum, and obliteration of the Douglas Pouch

According to the pathological examinations, bowel endometriotic lesions were classified into three sub-groups: sub-serosal layer involved, muscular layer involved, and mucous layer involved. In our current database, the sign of obliteration of the Douglas Pouch, there would be more chance to be detected for muscular and mucous layer involved (86.5% and 93.3%) than sub-serosal layer involved (74%). Compared to sub-serosal layer involved BE, we found that traction sign and thickening sign of the rectal wall were typical changes for muscular and mucous layer involved BE (Table 3). No notable differences were observed among other radiological features for different infiltrating depth. Utilizing the ROC analysis, the most favorable cutoff value for the thickness of the rectal wall corresponding to bowel endometriosis was 6.0 mm, with AUC was 0.89 (95% CI: 0.84–0.94, *p* < 0.01) (Fig. 6). For patients with the measured thickness of the rectal wall exceeding 6.0 mm, 72.1% (93/129) were confirmed BE with lesions infiltrated beyond the muscular layer of rectal wall.

**Table 3:** Sub-group analysis for different infiltrating depth of bowel endometriosis

Variables	Sub-serosal layer involved (*N* = 50)	Muscular layer involved (*N* = 89)	Mucous layer involved (*N* = 15)	P1	P2	P3
Anatomic location of the rectum				0.40	0.09	0.10
Upper	11	29	4			
Middle	37	56	8			
Lower	4	8	3			
Thickness of the rectal wall (mm)	6.70 ± 0.35	8.94 ± 0.44	14.84 ± 1.86	< 0.01	< 0.01	< 0.01
Traction sign of the rectum	28 (56%)	69 (77.5%)	13 (86.7%)	0.01	0.04	0.73
Obliteration of the Douglas Pouch	37 (74%)	77 (86.5%)	14 (93.3%)	0.11	0.16	0.69
Accompanied with adenomyosis	31 (62%)	63 (70.8%)	12 (80%)	0.35	0.55	0.23
Pelvic adhesion	47 (94%)	88 (98.9%)	15 (100%)	1.00	1.00	1.00

P1: comparison between patients with sub-serosal layer involved bowel endometriosis (BE) and muscular layer involved BE; P2: comparison between patients with muscular layer involved BE and mucous layer involved BE; P3: comparison between patients with sub-serosal layer involved BE and mucous layer involved BE

## Discussion

In this study, we depicted a novel method for reliably measuring thickness, traction sign of the rectum, and obliteration of the Douglas Pouch as well as pelvic adhesion from MRI data. Through the identification of relevant anatomic landmarks and rigorous classification of morphometric parameters, clear visualization of BE could be achieved.

Recently, MRI has been gradually applied as a crucial preoperative examinations for endometriosis [19], but the current diagnostic accuracy for BE has been queried. Reviewing previous studies, the overall sensitivity and specificity for detecting BE ranges widely from 63 to 98% [20–22]. Some of the studies only relied on radiological observations and the clinical diagnosis, while lacking pathological confirmation. Unlike rectal cancer, BE lesions appear relatively hypointense without obvious enhancement on T2WI, leading to a greater degree of occult disease. These relatively “dark” lesions of BE limit the detection capability, especially for less experienced radiologists. Previous study showed that clinical experience played an important role for diagnosing BE [23], with the AUC 0.862, 0.872, 0.744, and 0.648 respectively for experts (over 10-year experience in female imaging), fellows (half to 2 years), senior residents (7–24 weeks), and junior residents (less than 6 weeks) (*p* < 0.001).

Insufficient experience in dealing with BE patients and lack of standard or unified definition for image features of BE would be the most possible reasons hidden behind. Therefore, current debates on bowel involvement in patients with DE focus on the following aspects: (i) the typical signs of BE which could be easily recognized even by junior doctors; (ii) independent radiological parameters which could be reliable even when compared to pathological diagnosis of BE.

Firstly, several graphic features were precisely defined, including anatomic location of the rectum, adhesion sign of the rectum, traction sign of the rectum, and blockage of the Douglas Pouch. Based on our case-by-case analysis, we discovered that these MRI features could be verified by surgical findings and post-operative pathological examination, exhibiting substantial consistency. Moreover, the inter-reader agreement for these graphic features was quite high, with the kappa value exceeding 0.70 (Supplementary Table 2). It means the current precise analysis of the graphic features was reproducible and can be widely promoted.

Reviewing published papers, some graphic features had also been noticed by other radiologists when detecting deep endometriosis lesions. Early in 2010, Yoon et al. proposed the “mushroom cap” sign on T2WI images as a typical sign for diagnosing BE [24]. Similarly, Verela et al. proposed that the “fortune cookie sign” could be a typical MRI signs in predicting sigmoid endometriosis, which was characterized by the kinking of the segment affected [25]. Due to histopathological changes of hypertrophy for muscular layer, the thickness of the rectal wall is noticeably increased than in healthy volunteers. Moreover, the implants of endometriotic tissue have the potential to erode through inner layers and lead to fibrosis changes of the muscularis propria. Therefore, folding and thickening signs of the rectal wall presenting on MRI images would be typical changes for BE [26].

Hence, to recognize independent radiological parameters for BE pre-operatively, a comparative analysis was conducted between two groups (BE and non-BE). According to our current database, both univariate and multivariate analysis confirmed the thickness of rectal wall as a reliable MRI parameter to predict bowel endometriosis, with the OR 1.59 (95% CI: 1.29–1.96, *p* < 0.01).

However, besides BE, DE without lesions infiltrated to the rectal wall, thickening sign of the rectal wall would also be observed. Therefore, for better differentiated diagnosis, further scrutiny of the data was warranted. According to analysis of the ROC curve, we compared the sensitivity and specificity of the continuous variables. We found that the rectal wall thickness of 6.0 mm was associated with the highest Youden Index. The area under curve was up to 0.89 (95% CI: 0.84–0.94), which showed great consistency with the actual situation. The cutoff value yielded a sensitivity of 78.6% and specificity of 90.3%. The current result for the first time raised an appropriate “warning line” for alerting bowel involvement in DE patients. Similarly, in a 5-year retrospective analysis for the rectal endometriosis, Rousset et al. found that thickness of nodule over 14 mm was the independent predictive sign for full-layer infiltrating of bowel wall (OR = 0.945) [27].

Previous studies revealed that the impact angle was an independent MRI parameter that can determine whether a conservative or radical surgical approach should be taken in cases of DE. In 2018, after reviewing 52 BE cases who underwent MRI examination preoperatively, Perandini et al. revealed that combining the impact angle over 110.6° and the maximum lesion size over 32 mm, the positive predictive value was up to 87% for predicting conservative or radical bowel-related surgery [16]. In our study, we also found that traction sign was another distinctive sign for BE. For bowel endometriosis patients, traction sign of the rectum was more common to be detected in sagittal plane of MRI images [71.4% (110/154) VS 11.1% (8/72), *p* < 0.01]. Multivariate logistic regression confirmed the independent role of traction sign of rectum in predicting BE, with the OR: 0.24 (95% CI: 0.09–0.67, *p* < 0.01).

Some studies proposed an association between DE of the posterior compartment and adenomyosis [28, 29]. Our univariate analysis similarly indicates that the proportion of patients with co-occurring adenomyosis was slightly higher in the group of BE than in the non-BE, but there was no statistical significance (66.9% (103/154) VS 55.6% (40/72), *p* = 0.09). Because of the anatomic proximity, the close attachment of posterior uterus and the adjacent colon increased the risk of the obliteration of the Douglas Pouch. Our data showed that obliteration of the Douglas Pouch was a common morphological change to be presented on MRI images among BE patients, with the detection rate 81.8% (126/154), which was significantly higher than non-BE (23.6% (17/66), *p* < 0.01). Multivariate analysis further validated the independent value of this finding (OR = 0.17, 95% CI: 0.07–0.40, *p* < 0.01). Referring to previous studies, extrinsic adenomyosis is the leading cause of the obliteration of the Douglas Pouch. Endometriotic lesions in the posterior uterus were much more common to be infiltrated to the rectal wall [30].

Although the study is prospective, there were some limitations. The current findings were established based on data obtained from a single institution single, tertiary specialized center, which may lead to selective bias when screening the patients. Taking care of the cost of MRI scanning, pelvic DE patients acted as the controls, which limitedly draw more representative conclusion among patients suffering different types of endometriosis. Despite these limitations, our confidence in applying the current findings to local hospitals in order to provide visualized evidence for proper pre-operative decision-making remains unobstructed. In the future, multicenter studies with larger scale patients should be conducted to verify the clinical utility of the current findings.

Effective communication between radiologists and surgeons, facilitated by MRI images, is crucial for achieving a satisfactory surgical outcome. With the precise definition of the graphic features of posterior pelvic, it would be a high possibility to recognize BE pre-operatively even for less experienced doctors. When a high possibility for bowel involvement was predicted pre-operatively, MDT consultation consisting of general surgeons could be carried out preoperatively, which would be an effective way to decide the surgical strategies and to minimize postoperative complications. Most importantly, according to our current findings, it will be possible to be widely applied even in local hospitals, to improve the radiological diagnosis capability. Therefore, peri-surgical decision-making for gynecologists will be elevated.

## Conclusion

In conclusion, thickening and traction signs of the rectum, as well as the obliteration of the Douglas Pouch, are the typical triad for BE. The rectal wall thickness exceeding 6 mm strongly suggests the presence of BE. Precise definition of radiological features allows for early detection of BE, which is crucial for preventing the progression timely and accelerating the diagnostic efficacy.

### Supplementary information

Below is the link to the electronic supplementary material.

Supplementary file1 (PDF 388 KB)

## Abbreviations

95% CI: 95% Confidence interval
AUC: Area under curve
BE: Bowel endometriosis
DE: Deep endometriosis
ESHRE/ESGE: European Society of Human Reproduction and Embryology/European Society for Gynecological Endoscopy
ESUR: European Society of Urogenital Radiology
MDT: Multidisciplinary treatment
MRI: Magnetic resonance imaging
OR: Odds ratio
PACS: Picture archiving and communication system
ROC: Receiver operating characteristic
T1WI: T1-weighted imaging
T2-FS: T2-fat suppression sequence
T2WI: T2-weighted imaging
VAS: Visual analog scale
YI: Youden index

## Declarations

### Guarantor

The scientific guarantors of this publication are Dr. Congjian Xu, and Dr. Xiaofang Yi, professors of Fudan University, focusing on the clinical research of endometriosis.

### Statistics and biometry

Dr. Jiangnan Wu, the expert in statistics of Obstetrics and Gynecology Hospital of Fudan University, kindly provided statistical advice for this manuscript.

### Study subjects or cohorts overlap

This was original research, and we confirmed that no study subjects or cohorts have been previously reported.

### Methodology

- prospective
- diagnostic or prognostic study/observational/experimental
- performed at one institution
