# Supplementary material for: Bowel wall thickness measured by MRI is useful for early diagnosis of bowel endometriosis
Source: Eur Radiol. 2023 Jul 27;33(12):9244–53. doi: 10.1007/s00330-023-09795-7 (PMC10667399; doi:10.1007/s00330-023-09795-7)

Bowel Wall Thickness Measured by MRI is Useful for Early Diagnosis of Bowel Endometriosis

ELECTRONIC SUPPLEMENTARY MATERIAL

Supplementary Table 1. MRI scanning parameters for different sequences

Parameters	TR （ms)	TE(ms)	FOV (mm)	Thickness (mm)	Voxel size (mm)	SNR
**T2WI-sag-FS**	4490	83	270	4.0	1.1*1.1*4.0	1.0
**T2WI-tra-FS**	8760	83	390	4.0	1.5*1.5*4.0	1.11
**T1WI-tra**	940	10	390	4.5	1.6*1.2*4.5	1.24
**T1WI-FS-tra**	4.89	2.38	380	5.0	2.0*1.2*5.0	1.0
**DWI**	3600	83	300	5.0	2.7*1.9*5.0	1.0
**DCE（sag）**	463	10	270	4.0	1.4*0.8*4.0	1.0
**DCE（tra）**	4.89	2.38	400	4.0	1.8*1.3*4.0	1.0

T1WI: T1-weighted image; T2WI: T2-weighted image; FS-T2WI: fat saturated T2-weighted image; DWI: diffusion weighted image; DCE: dynamic contrast enhancement; sag: sagittal position; tra: transverse position; SNR: signal noise ratio; TR: time of repetition; TE: time of echo; FOV: field of view.

Supplementary Table 2. Reliability analysis of intra-readers.

Factors	Kappa Coefficient	95%CI
**Thickness of the rectal wall**	0.79	0.70-0.87
**Traction sign of rectum**	0.85	0.78-0.92
**Obliteration of the Douglas Pouch**	0.81	0.73-0.89
**Sign of adenomyosis**	0.74	0.65-0.83
**Pelvic adhesion**	0.70	0.56-0.82

Supplementary Figure 1. A typical case of bowel endometriosis. (a) Sagittal plane of pelvis, DE lesion was marked with red arrow (→). (b) Colonoscopy showed a solid mass (*) infiltrated to rectum wall. (c) Laparoscopic view of the BE lesions. (D) Gross examination of the specimen. Histological findings showing the endometrial gland invaded into the mucosal layer with HE staining at 10x magnification.

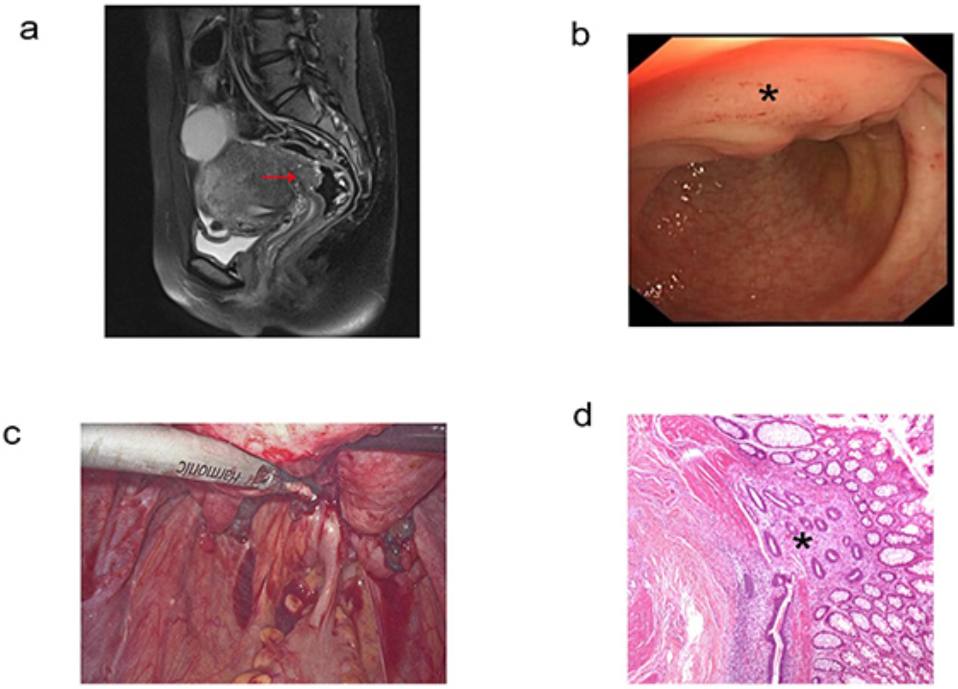

Supplement: Supplementary file 1 — Supplementary file1 (PDF 388 KB) [file 330_2023_9795_MOESM1_ESM.pdf]
